# Antibiotic safety among neonates and paediatrics in a public hospital: KwaZulu-Natal

**DOI:** 10.4102/hsag.v28i0.2464

**Published:** 2023-12-20

**Authors:** Tyler A. Frank, Frasia Oosthuizen, Varsha Bangalee

**Affiliations:** 1 Discipline of Pharmaceutical Sciences, College of Health Sciences, University of KwaZulu-Natal, Durban, South Africa

**Keywords:** antibiotics, paediatrics, neonates, drug safety, rational use, broad spectrum

## Abstract

**Background:**

The World Health Organization (WHO) guidelines recommend the empiric treatment of infections before definitive treatment begins. However, ethical concerns limit the availability of clinical trials in neonates and paediatrics to fully ascertain the safety profile of antibiotics in these populations.

**Aim:**

This study aimed to quantify the use of antibiotics among neonates and paediatrics and commented on the use, rationale and appropriateness of antibiotics prescribed.

**Setting:**

A secondary level public sector hospital located in Durban, KwaZulu-Natal.

**Methods:**

Demographic and treatment information of neonates and paediatrics were collected retrospectively from January 2022 to June 2022. Data were obtained from patient files and extracted for analysis using Microsoft Excel^®^. Analytical and descriptive statistics were used to analyse patient demographics and treatment variables.

**Results:**

A total of 568 antibiotics, issued to 389 patients, were reviewed. Penicillins (40.1%), aminoglycosides (24.3%) and combination penicillin-beta-lactam inhibitors (23.3%) were identified as the most frequently prescribed antibiotics for inpatients. Most antibiotics prescribed to inpatients were for complications associated with pre-term birth (66.9%). Combination penicillin-beta-lactam inhibitors (34.7%), penicillins (29.5%) and cephalosporins (29.5%) were the most frequently prescribed antibiotics to outpatients. A correlation was found between the route of administration and the duration of therapy; the intravenous route (63.6%) was preferred over the oral route (36.4%) for administration.

**Conclusion:**

Many broad-spectrum antibiotics were prescribed, thus increasing the risk of resistance. Antibiotics were being prescribed according to the guidelines; however, there is still a need for therapeutic drug monitoring to ensure the continuation of rational drug use.

**Contribution:**

There was evidence of rational use of antibiotics in the public hospital (KwaZulu-Natal), in keeping with economic and availability factors.

## Introduction

Antimicrobials are the most prescribed drug class in the neonatal and paediatric community, where they play an integral role in the treatment of infections (Coon et al. 2017). The World Health Organization (WHO) guidelines recommend the empiric treatment of infections before definitive treatment begins (WHO 2016). Empiric treatment is the treatment of an infection before the causative agent is known and involves the use of broad-spectrum antibiotics, especially in developing countries because of the limited amount of available medical resources (WHO 2016).

Many neonatal deaths arise from infections, including sepsis, tetanus, pneumonia and diarrhoea (Thaver & Zaidi 2009). Neonates and paediatrics are at risk of infections because of the immaturity of their immune systems, as well as increased exposure to infections during hospital admissions. In developing countries, the burden of infection is exacerbated by the below-optimal living conditions (Thaver & Zaidi 2009). The risk of contracting infections is further heightened in African countries because of the high incidence of human immunodeficiency virus (HIV), affecting a person’s immunity (Olivier et al. 2018). The use of antimicrobials in the neonatal and paediatric population is therefore unavoidable (Van den Anker & Allegaert 2019).

The empiric treatment of infections mainly involves the use of broad-spectrum antibiotics (Naidoo et al. 2021). Antibiotic use can result in an alteration in the microorganisms that reside in the body, having dire effects on the developing immune system (Cotten 2016). Furthermore, the incorrect use of antibiotics can result in antimicrobial resistance (Chrichton et al. 2018). Antimicrobial resistance has a significant impact on patient outcomes, resulting in higher mortality rates, prolonged hospitalisation and an increase in the economic burden (Folgori & Bielicki 2019).

The WHO defines the rational use of medicines as:

[*P*]atients receiving medications appropriate to their clinical needs, in doses that meet their own individual requirements, for an adequate period of time, and at the lowest cost to them and their community. (WHO 2002)

In the context of this study, the rational use of antibiotics refers to the appropriateness of the antibiotic, in the most appropriate dosage form, prescribed in the correct doses, specific to the medical condition of the neonate or paediatric. The unnecessary exposure of neonates and paediatrics to antibiotics increases the risk of serious adverse events, increased healthcare costs, and is responsible for the significant rise in antimicrobial resistance (Shehab et al. 2008).

The unnecessary exposure of antibiotics places patients at risk of adverse events such as antibiotic resistance (Centers for Disease Control and Prevention 2023). Antibiotic resistance continues to remain a serious threat to public health and is attributed to the overuse of antibiotics, inappropriate prescribing and the unavailability of new antibiotics in the healthcare setting (Ventola 2015). Antibiotic stewardship has been defined as:

[*C*]oordinated interventions designed to improve and measure the appropriate use of [antibiotic] agents by promoting the selection of the optimal [antibiotic] drug regimen including dosing, duration of therapy, and route of administration. (Barlam et al. 2016)

Antibiotic stewardship programmes are put into place to optimise the use of antibiotics, reducing antibiotic resistance, improving patient outcomes, decreasing the spread of infections caused by resistant organisms and decreasing healthcare costs (Centers for Disease Control and Prevention 2023). Currently, antimicrobial stewardship, together with infection prevention and control, and medicine and patient safety, form an integrated approach to health systems strengthening (WHO 2019).

The lack of clinical trials in the paediatric population has resulted in limited data on the safety and use of antibiotics in paediatrics. Paediatric clinical trials differ from adult clinical trials because of the legal and ethical issues around the consent of the child, the need for age-appropriate study information, limited sample sizes, and safety issues related to the development and maturation of the study population (Meyers et al. 2020). Therefore, the effects of these drugs on the neonatal and paediatric population can only be verified after its use in clinical practice (Alomar 2014). However, approaches such as mathematical modelling have been used in this regard to provide statistical models of antibiotic use and resistance. The use of mathematical modelling results in more information about antibiotic use in the paediatric population, providing a sense of guidance to prescriber decision-making (Olesen 2022). The quantification and evaluation of antibiotics among this population allows for the identification of prescribing patterns as well as for the review of current safety data (Naidoo et al. 2021).

In response to the aforementioned issue, this study makes use of current data to determine appropriate and rational prescribing techniques in hospitals. The qualification and quantification of antibiotic use in neonates and paediatrics is described and parameters such as gender and age distribution, duration of hospital stay, most commonly prescribed antibiotics and the prescribing pattern are investigated. The primary aim of this study was therefore to investigate the use, rationale and appropriateness of antibiotics administered to neonates and paediatrics in a public healthcare facility (KwaZulu-Natal, South Africa). This was achieved through the following objectives:

- To quantify antibiotic use (type, dose, formulation, therapeutic indication) in neonates and paediatrics in a public healthcare facility (King Dinuzulu Hospital Complex)
- To comment on the rationale of antibiotics used
- To determine if the drug choice and dosage form is appropriate for the diagnosis and the patient
- To review the safety profile of antibiotics used in neonates and paediatrics based on current literature

## Methods

### Study design

A retrospective observational study was performed to evaluate antibiotic use among neonates and paediatrics in a public sector hospital (King Dinuzulu Hospital Complex). The quantitative study determined the rational use of antibiotics by evaluating antibiotic use (type, dose, formulation, therapeutic indication) within the study population.

### Setting

This retrospective study was conducted over a 6-month period (January 2022 to June 2022) at a secondary level public sector hospital (King Dinuzulu Hospital Complex), centrally located in Durban, KwaZulu-Natal, South Africa.

### Study population and sampling strategy

The study population consisted of both inpatients, admitted to the ward, and outpatients, receiving medication from the pharmacy after a consultation with a medical practitioner. The following inclusion criteria were applied to select the study population:

- 18 years of age or younger; this included neonates of 0–1 month, preterm babies and paediatric patients, (i.e. older than 1 month to 18 years of age)
- A script containing at least one antibiotic
- Inpatient or outpatient

Exclusion criteria:

- Older than 18 years of age
- No antibiotic prescribed

A total of 389 patient files met the inclusion criteria over the 6-month period the study was conducted. This sample size was large enough to allow for a comprehensive analysis of antibiotic usage.

### Data collection

Data were obtained from patient files after permission and ethical approval was granted. No identifiable information was extracted from patient files. A predesigned data extraction tool, using Microsoft Excel^®^, was used to record the available patient demographic and antibiotic treatment information (Appendix 1). The template was created and guided by the requirements of the WHO. It included essential antimicrobial use indicators that were designed specifically for data collection in hospitals (USAID 2012).

The following data were extracted:

- Demographics (age, ethnicity, gender and HIV status)
- Antibiotic(s) prescribed
- Dosage of antibiotics
- Route of administration of antibiotics
- Frequency of administration of antibiotics
- Duration of treatment of antibiotics
- Diagnosis

The collected data were kept confidential and stored on a password-controlled computer. Upon complete interpretation and dissemination of results, the collected data were removed. Individual patients’ written informed consent was not needed, as only anonymised, retrospective data were extracted.

### Data analysis

Analytical and descriptive analysis was used to describe patient demographic information and antibiotic treatment variables (Naidoo et al. 2021). Patterns in antibiotic usage were identified through the use of statistical measures, such as frequency and mean. The quantitative data values were grouped into categories and then represented in frequency distribution tables as well as in a frequency distribution bar chart (Naidoo et al. 2021). The relationship between duration of treatment, antibiotic type and course duration was compared between inpatients and outpatients; however, no comparison could be made because different conditions were being treated in the two groups.

The use of a probability sampling method, which included all eligible participants who visited the hospital during the period of January 2022 to June 2022, eliminated the probability for biased selection. The probability for biased selection was further eliminated because data were collected and extracted by the researcher only, with the use of a predesigned tool (Naidoo et al. 2021).

### Ethical considerations

Ethical approval was obtained from the Biomedical Research Ethics Committee (BREC) at the University of KwaZulu-Natal (UKZN) (ethical approval number: BREC/00003684/2021), KZN Department of Health (KZN-DoH), and Chief Executive Officer (CEO) at King Dinuzulu Hospital Complex.

## Results

### Demographic and general characteristics

Patient demographic data for the 389 neonatal and paediatric patients included in the study are shown in Table 1. Most of the study participants (79.4%) were between the ages of 0 and 24 months, with 46% being inpatients and 33.4% being outpatients. Almost 87% (86.6%) of the study population were African, with a small percentage being Indian (7.5%), mixed race (3.3%) and white (2.8%) population. A small percentage of the study participants were co-infected with HIV (11.8%), of which 13% were inpatients and 3.3% outpatients.

**TABLE 1:** Characteristic and demographic data of study population.

Demographics	Frequency (*N*)		%	
	Inpatients	Outpatients	Inpatients	Outpatients
**Age (months)**				
0–24	179	130	46.0	33.4
> 24	5	75	1.3	19.3
**Race**				
Black people	162	175	41.6	45.0
Asian people	8	20	2.1	5.1
Mixed race people	8	5	2.1	1.3
White people	6	5	1.5	1.3
**HIV co-infection**				
Yes	33	13	8.5	3.3
No	151	192	38.8	49.4
**Inpatient days**				
1–3	83	0	21.3	-
4–10	88	0	22.6	-
11–28	13	0	3.3	-
**Outpatient visits**				
1	0	203	-	52.2
2–3	0	2	-	0.5

A list of the number of prescribed antibiotics per class and the number of prescriptions is shown in Table 2. A total of 568 antibiotics were dispensed to 389 patients. Most patients (88.4%) received one antibiotic, with only 2.6% of patients receiving more than two antibiotics.

**TABLE 2:** The prescribed antibiotic classes and respective antibiotics.

Antibiotic class	Antibiotic prescribed	Frequency	%	Total doses prescribed
Penicillins	Amoxicillin	74	13.0	1308
	Ampicillin	94	16.5	1106
	Benzathine benzylpenicillin	31	5.5	306
	Cloxacillin	1	0.2	1
	Flucloxacillin	1	0.2	24
	Total	201	35.4	2745
Combination penicillin and beta-lactam inhibitors	Amoxicillin/clavulanic acid	135	23.8	1596
	Piperacillin/tazobactam	26	4.6	234
	Total	161	28.3	1830
	Total penicillin use	362	63.7	4575
Cephalosporins	Ceftriaxone	71	12.5	71
	Cefotaxime	17	3.0	189
	Cephalexin	2	0.4	2
	Total	90	15.9	262
Aminoglycosides	Amikacin	27	4.7	81
	Gentamycin	51	9.0	301
	Total	78	13.7	382
Nitroimidazoles	Metronidazole	14	2.5	162
	Total	14	2.5	162
Macrolides	Azithromycin	12	2.1	48
	Total	12	2.1	48
Anti-tuberculosis agents	Isoniazid	5	0.9	694
	Total	5	0.9	694
Fluoroquinolones	Ciprofloxacin	4	0.7	26
	Total	4	0.7	26
Beta-lactam carbapenems	Meropenem	3	0.5	27
	Total	3	0.5	27
	Total	568	100	6176

Penicillins were most frequently prescribed (35.4%), followed by cephalosporins (15.9%) and aminoglycosides (13.8%). Other antibiotics such as nitroimidazoles (2.5%), macrolides (2.1%), anti-tuberculosis antibiotics (0.9%), fluoroquinolones (0.7%) and carbapenems (0.5%) were least prescribed.

Table 2 also includes the total number of doses prescribed for each antibiotic class. Penicillin-containing antibiotics contributed to majority of the antibiotics that were dispensed to patients.

A total of nine antibiotic classes and their dosing schedules, including the route of administration and indications for use, are described in Table 3. Penicillins were the most frequently prescribed antibiotic class (40.1%), with ampicillin being the most frequently prescribed penicillin (72.4%). In the aminoglycoside class, gentamycin was most frequently prescribed (64.9%). Macrolides and fluoroquinolones were present in very small percentages, with azithromycin being the only prescribed macrolide and ciprofloxacin being the only prescribed fluoroquinolone.

**TABLE 3:** Characteristics of antibiotic classes prescribed for neonatal and paediatric patients admitted to the hospital.

Antibiotic class Class	Description	Strength	Dosing frequency	Average duration of treatment (days)	Route of administration	Indication for use	Number of prescriptions (*N* = 317)
Penicillins	Amoxicillin	125 mg/5 mL; 250 mg/5 mL	8 hourly	4	Oral	Complications of the respiratory system Complications of preterm birth†	4
	Ampicillin	250 mg; 500 mg	6–12 hourly	4	IV‡	-	92
	Benzathine benzylpenicillin	600 000 IU/mL	Once immediately§	-	IM¶	-	6
			6–12 hourly	4	-	-	25
	Total	-	-	-	-	-	127
Aminoglycosides	Amikacin	100 mg/2 mL	24 hourly	3	IV	Complications of preterm birth	27
	Gentamycin	10 mg/mL; 20 mg/2 mL; 40 mg/mL; 80 mg/2 mL	6-12 hourly	3	IV	-	50
	Total	-	-	-	-	-	77
Combination of penicillins and beta-lactam inhibitors	Amoxicillin clavulanic acid	125 mg/5 mL 250 mg/5 mL	Once immediately	-	Oral; IV	Complications of the respiratory system	13
			0–24 hourly	4		-	35
	Piperacillin/Tazobactam	0.5 g/4 g	6–8 hourly	3	IV	-	26
	Total	-	-	-	-	-	74
Cephalosporins	Ceftriaxone	500 mg;1 g	Once immediately	-	IV	Complications of the respiratory system	2
	Cefotaxime	500 mg	8–12 hourly	3	IV		14
	Total	-	-	-	-	-	16
Nitroimidazoles	Metronidazole	200 mg/5 mL; 500 mg/100 mg	8–24 hourly	3	Oral; IV	Complications of the gastrointestinal system	10
	Total	-	-	-	-	-	10
Macrolides	Azithromycin	200 mg/5 mL	24 hourly	4	Oral	Complications of preterm birth	7
	Total	-	-	-	-	-	7
Beta-lactam carbapenems	Meropenem	500 mg	8 hourly	3	IV	Complications of preterm birth and no response to other antibiotics††	3
	Total	-	-	-	-	-	3
Fluoroquinolones	Ciprofloxacin	2 mg/mL	12 hourly	2	IV	Complications of preterm birth and no response to other antibiotics	2
	Total	-	-	-	-	-	2
Anti-tuberculosis agents	Isoniazid	50 mg/mL	24 Hourly	22	Oral	TB prophylaxis	1
	Total	-	-	-	-	-	1

IV, Intravenous injection; IM, Intramuscular injection.

† , Complications of preterm birth include conditions such as prematurity, congenital or neonatal sepsis, premature rupture of membranes, perinatal asphyxia, foetal distress and congenital syphilis;

‡ , All doses administered as once immediately were only administered as single, immediate doses. No further doses were administered;

§ , No response to penicillin-containing antibiotics – switched to alternatives because of antimicrobial resistance;

¶ , All doses administered as once immediately were only administered as single, immediate doses. No further doses were administered;

†† , No response to penicillin-containing antibiotics – switched to alternatives because of antimicrobial resistance.

Doses of the prescribed antibiotics were adjusted according to the body weights of the neonates and paediatrics. Because there were a large number of neonates (66.9%) who presented with complications associated with preterm birth, the body weight of the neonate was integral in determining the appropriate antibiotic dose.

Most inpatients received antibiotics intravenously (IV), while benzylpenicillin was administered intramuscularly (IM). Majority of the antibiotics were given for 5 days or less (89.7%). Isoniazid was an exception as the average duration of treatment for this antibiotic was 22 days.

Table 4 describes the dosing characteristics per antibiotic class. Combination penicillins (34.7%), penicillins (29.5%) and cephalosporins (29.5%) were the three most frequently prescribed antibiotic groups. Amoxicillin was the most prescribed penicillin (94.6%). Azithromycin was the only prescribed macrolide and ciprofloxacin was the only prescribed fluoroquinolone. Azithromycin was prescribed for the treatment of interstitial pneumonia and respiratory distress associated with prematurity of the lungs. This antibiotic is included on the Essential Medicines List in the public health sector.

**TABLE 4:** Characteristics of antibiotic classes prescribed for outpatients.

Antibiotic class	Description	Strength	Dosing frequency	Average duration (days)	Route of administration	Common indication by disease category	Number of prescriptions (*N* = 251)
Combination of penicillins and beta-lactam inhibitors	Amoxicillin/clavulanic acid	125 mg/5 mL; 250 mg/5 mL; 500 mg/100 mg	Once immediately†	-	Oral; IV	Complications of the respiratory system	23
			6–24 hourly	6	-	-	64
	Total	-	-	-	-	-	87
Penicillins	Amoxicillin	125 mg/5 mL; 250 mg/5 mL	8 hourly	6	Oral	Complications of the respiratory system Disorders of the skin	70
	Ampicillin	250 mg; 500 mg	Once immediately	-	IV	-	2
	Cloxacillin	500 mg	Once immediately	-	IM	-	1
	Flucloxacillin	250 mg	6 hourly	6	Oral	-	1
	Total	-	-	-	-	-	74
Cephalosporins	Ceftriaxone	500 mg;1 g	Once immediately	-	IV	Complications of the respiratory system Disorders of the skin	69 3 2
	Cefotaxime	500 mg	8–12 hourly	7	Oral	-	
	Cephalexin	250 mg/5 mL	Once immediately	-	IV	-	
	Total	-	-	-	-	-	74
Macrolides	Azithromycin	200 mg/5 mL	24 hourly	4	Oral	Complications of the respiratory system	5
	Total	-	-	-	-	-	5
Nitroimidazoles	Metronidazole	200 mg/5 mL; 500 mg/100 mg	8–24 hourly	6	Oral	Complications of the gastrointestinal system	4
	Total	-	-	-	-	-	4
Anti-tuberculosis agents	Isoniazid	50 mg/mL	24 hourly	168	Oral	TB prophylaxis	4
	Total	-	-	-	-	-	4
Fluoroquinolones	Ciprofloxacin	2 mg/mL	12 hourly	5	IV	Complications of the respiratory system	2
	Total	-	-	-	-	-	2
Aminoglycosides	Gentamycin	80 mg/2 mL	Once immediately	-	IV	Complications of the respiratory system	1
	Total	-	-	-	-	-	1

IV, Intravenous injection; IM, Intramuscular injection.

† , All doses administered as once immediately were only administered as single, immediate doses. No further doses were administered.

A total of 68.1% of the antibiotics were prescribed for periods equal to or longer than 5 days. Cephalexin was the only agent prescribed for an average period of 7 days. Isoniazid was an exception in this group, with the average duration of treatment being 168 days for a total of four patients. Most patients received either an oral or intravenous dose of administration.

**FIGURE 1:**
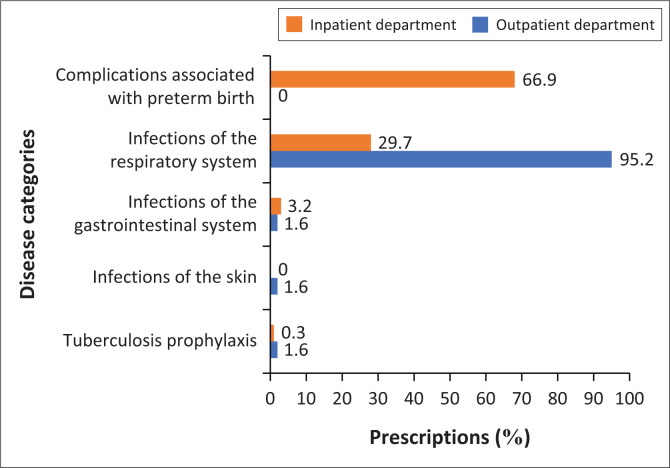
The percentage of prescriptions allocated to each disease category for inpatients and outpatients.

Figure 1 shows the indications of antibiotics for inpatients and outpatients separately. Most of the inpatient prescriptions were for complications associated with preterm birth (66.9%), i.e. babies born alive before 37 weeks of pregnancy are completed, followed by infections of the respiratory system, e.g. pneumonia and bronchitis (29.7%). For inpatients, gastrointestinal infections, e.g. gastroenteritis, dysentery (3.2%) and tuberculosis prophylaxis (0.3%) contributed to a smaller number of prescriptions. Most of the prescriptions for outpatients were for infections of the respiratory system (95.2%). Skin infections (1.6%), gastrointestinal infections (1.6%) and tuberculosis prophylaxis (1.6%) contributed equally for the remainder of the prescriptions.

## Discussion

This study aimed to quantify and evaluate the use and appropriateness of antibiotics in neonates and paediatrics and to comment on the rationale for use. A comparison was made between treatment data collected from the patient files and the Standard Treatment Guidelines (Standard Treatment Guidelines and Essential Medicines List for South Africa, Hospital Level Paediatrics 2020), as well as the South African Medicines Formulary (Rossiter, Blockman, Barnes [eds.], University of Cape Town Division of Clinical Pharmacology, South African Medical Association, Health and Medical Publishing Group 2020). The rational use of antibiotics in the neonatal and paediatric population was measured by evaluating the treatment regimen and determining if the correct drug was used for the condition diagnosed, in the correct doses and using the correct route of administration.

The prescribing of an antibiotic to a specific patient is based on the safety and side effect profile, as well as the pathogen and resistance pattern (Van den Anker & Allegaert 2019). This is especially important in the treatment of paediatric patients and neonates because the physiological differences in the body of a child, compared with an adult, will influence the pharmacokinetic and pharmacodynamic parameters of the antibiotic (Al-Metwali & Mulla 2017). As there is a vast amount of evidence-based information on the use of penicillins, this antibiotic class is widely used in neonates and paediatrics (Van den Anker & Allegaert 2019).

Like other studies described in the literature, more than a third of the study population received a penicillin antibiotic. This category of antibiotics remains widely prescribed as empiric treatment for infections in neonates and paediatrics (Van den Anker & Allegaert 2019). Amoxicillin was the most prescribed penicillin; this is because of the broad spectrum of amoxicillin and the fact that this drug is on the Essential Medicines List for the public health sector (Standard Treatment Guidelines and Essential Medicines List for South Africa, Hospital Level Paediatrics 2020).

In the study conducted, there was empiric treatment of infections with a broad-spectrum antibiotic before further investigation of the pathogen was conducted. This is because of the lack of resources in a public facility. Further investigation of the pathogen and resistance pattern of the patient was only conducted if the patient showed no response to the treatment prescribed. This observation was only seen in the inpatient department.

Treatment duration with penicillin antibiotics ranged from 4 to 6 days and was generally within the normal prescribing guidelines (South African Medical Formulary – 13th edn.). Because the study was based on the neonatal and paediatric population, doses were adjusted according to the weights of the neonates and paediatrics. This observation was especially seen in the neonatal population of the inpatient department, because a large percentage of neonates presented with complications associated with preterm birth.

The transition from the sheltered intrauterine environment to the bacteria-filled external world is significant, even more so when neonates are born preterm (Tsafaras et al. 2020). The adjustment of the preterm neonate is an essential factor that influences antibiotic therapy (Greenberg et al. 2019). It is therefore imperative to introduce antibiotic therapy to preterm infants within 24 h of birth to prevent the risk of mortality because of early-onset sepsis. The administration of broad-spectrum antibiotics to the population of preterm infants was seen to be successful in limiting mortality associated with early-onset sepsis (Greenberg et al. 2019).

Empiric antibiotics are essential for preterm infants to prevent the occurrence of early-onset sepsis (Greenberg et al. 2019). For this reason, a large percentage of penicillin and combination penicillin antibiotics were prescribed. Early-onset sepsis normally manifests as pneumonia, with respiratory symptoms being common in both full-term and preterm neonates (Manan et al. 2014). Penicillin and combination penicillin antibiotics are one of the most widely used empiric antibiotics because of their effectiveness against an extensive range of infections involving both gram-positive and gram-negative cocci, gram-positive rods and most anaerobes. The broad spectrum of penicillin and penicillin-containing antibiotics make it an ideal candidate for the empiric treatment of infections (Friedland & McCracken 1994).

In this study, bacterial resistance was cited as the reason for switching patients from penicillin-containing antibiotics to fluoroquinolones or beta-lactam carbapenems in patients who were admitted as inpatients. The use of carbapenems is reserved for bacterial infections that show resistance to penicillins and broad-spectrum beta-lactam antibiotic therapy (Folgori & Bielicki 2019). The use of fluoroquinolone or carbapenem antibiotics in the study population was minimal, indicating that this category of drug therapy was reserved for use if no other antibiotic was viable for treatment (Walsh & Wright 2005).

Ciprofloxacin, an antibiotic used for infections of the urinary system, is sparsely used in the neonatal and paediatric populations. This is because of the musculoskeletal adverse effects of this drug. These musculoskeletal adverse effects include arthropathy, which is described as diseases of the joints, and arthralgia, which is described as stiffness of the joints (Adefurin et al. 2011). According to a systematic review of the use of ciprofloxacin in paediatric patients, all musculoskeletal adverse effects are reversible following the withdrawal of the drug (Adefurin et al. 2011).

No correlation was found between the age of the patients and the duration of antibiotic treatment. However, it was seen that patients admitted to the hospital had a shorter antibiotic treatment duration compared with patients in the outpatient department. This correlation could be related to the route of administration of the antibiotics, as patients admitted to the hospital were administered IV antibiotics. When drugs are administered via the intravenous route, the drug reaches peak concentration in a shorter time, resulting in faster therapeutic outcomes (Jin et al. 2015). In the outpatient department, patients received oral antibiotics, resulting in the drug taking a longer time to reach peak plasma concentrations, hence the longer duration of treatment (Jin et al. 2015).

The use of isoniazid was seen in a minute percentage (0.9%) of the study population. Isoniazid use in this population is not uncommon because of the risk of tuberculosis. Tuberculosis among paediatric patients is common in TB burden countries, with childhood tuberculosis contributing to 20% of all tuberculosis cases in South Africa (Zar et al. 2012). There have been vast advancements in the research of childhood tuberculosis, highlighting prophylaxis as a major factor in curbing the spread of childhood tuberculosis (Tadesse et al. 2016). The Centers for Disease Control and Prevention advise the use of isoniazid as prophylaxis for all infants, young children and immunocompromised children who have been exposed to someone with infectious TB disease (Centers for Disease Control and Prevention 2016).

According to several studies, most antibiotics are administered for respiratory tract infections or gastroenteritis (Alter et al. 2011; Alharbi et al. 2022). This study had similar findings. Skin infections and infections of the gastrointestinal system were seen in minute percentages. Antibiotics used for the empiric treatment of infections, such as penicillin-containing antibiotics, cephalosporins, macrolides and aminoglycosides, have been shown to be effective in neonates and paediatrics (Van den Anker & Allegaert 2019). According to the patient files, laboratory tests were only conducted for inpatients; there were no laboratory test findings included in patient files obtained from the paediatric outpatient department.

## Limitations

The study was conducted in one hospital site, in one city, and only included patients who visited the hospital from January 2022 to June 2022, with a moderate study population. Therefore, it cannot be assumed that these results are extrapolated to either the national population (public/private sector) or the general South African population. The limitation also exists in the patients’ demographic data that could be extracted from patients’ records. Despite these limitations, this study contributes to research in this population group and builds on the evidence that currently exists.

## Implications or recommendations

There is still a need for further research to be conducted in the paediatric and neonatal population and for the implementation of mathematical modelling in the prescribing techniques of South African healthcare professionals. Healthcare professionals across the board need to keep abreast with newly published safety data on antibiotics, thereby improving their prescribing practices ensuring the safe use of antibiotics in the neonatal and paediatric populations. There remains a need for therapeutic drug monitoring for certain antibiotics (e.g., aminoglycosides, vancomycin) under certain conditions to ensure the continuation of rational drug use.

## Conclusion

This study shows that, despite the limitations in clinical evidence, there are many categories of antibiotics that are used in the neonatal and paediatric populations. Rational use of antibiotics in neonates and paediatrics describes the prescribing and administering of a safe formulation of the optimal dose based on clinical decision-making and evidence-based research. This study found that there is a rational use of antibiotics in the aforementioned public healthcare facility (KwaZulu-Natal, South Africa), in keeping with economic and availability factors. It has been shown that there is empiric treatment of infections before definitive treatment begins, attributing to the large penicillin use. However, there is safe medicine use that is occurring, as antibiotic dosages as well as dosing schedules are aligned with the Standard Treatment Guidelines.

## Appendix 1

Date: _____________________________________

**TABLE A1:** Data collection template.

**Demographics**	-	-	-	-	-	-	-	-	-	-	-
Age	-	-	-	-	-	-	-	-	-	-	-
Ethnicity	-	-	-	-	-	-	-	-	-	-	-
HIV co-infection	-	-	-	-	-	-	-	-	-	-	-
Inpatient days	-	-	-	-	-	-	-	-	-	-	-
Outpatient visits	-	-	-	-	-	-	-	-	-	-	-
**Investigative data**	-	-	-	-	-	-	-	-	-	-	-
Antibiotic name	-	-	-	-	-	-	-	-	-	-	-
Dose/dosing frequency	-	-	-	-	-	-	-	-	-	-	-
Duration of treatment	-	-	-	-	-	-	-	-	-	-	-
Route of administration	-	-	-	-	-	-	-	-	-	-	-
Diagnosis	-	-	-	-	-	-	-	-	-	-	-
Total number of prescriptions	-	-	-	-	-	-	-	-	-	-	-
